# ZnO/γ-Fe_2_O_3_/Bentonite: An Efficient Solar-Light Active Magnetic Photocatalyst for the Degradation of Pharmaceutical Active Compounds

**DOI:** 10.3390/molecules27103050

**Published:** 2022-05-10

**Authors:** Mohammadreza Kamali, Yongtao Xue, Mohammadreza Khalaj, Bodhi Laats, Ruben Teunckens, Matthias Verbist, Maria Elisabete V. Costa, Isabel Capela, Lise Appels, Raf Dewil

**Affiliations:** 1 Process and Environmental Technology Lab, Department of Chemical Engineering, KU Leuven, J. De Nayerlaan, 5, 2860 Sint-Katelijne-Waver, Belgium; mohammadreza.kamali@kuleuven.be (M.K.); yongtao.xue@kuleuven.be (Y.X.); bodhi.laats@student.kuleuven.be (B.L.); ruben.teunckens@student.kuleuven.be (R.T.); matthias.verbist@student.kuleuven.be (M.V.); lise.appels@kuleuven.be (L.A.); 2 Department of Environment and Planning, Center for Environmental and Marine Studies, CESAM, University of Aveiro, 3810-193 Aveiro, Portugal; khalaj@ua.pt (M.K.); icapela@ua.pt (I.C.); 3 Department of Materials and Ceramics Engineering, Aveiro Institute of Materials, CICECO, University of Aveiro, 3810-193 Aveiro, Portugal; elisabete.costa@ua.pt

**Keywords:** engineered photocatalytic nanomaterials, solar irradiation, pharmaceutical active compounds, mechanistic studies

## Abstract

For applications related to the photocatalytic degradation of environmental contaminants, engineered nanomaterials (ENMs) must demonstrate not only a high photocatalytic potential, but also a low tendency to agglomeration, along with the ability to be easily collected after use. In this manuscript, a two-step process was implemented for the synthesis of ZnO, ZnO/Bentonite and the magnetic ZnO/γ-Fe_2_O_3_/Bentonite nanocomposite. The synthesized materials were characterized using various techniques, and their performance in the degradation of pharmaceutical active compounds (PhACs), including ciprofloxacin (CIP), sulfamethoxazole (SMX), and carbamazepine (CBZ) was evaluated under various operating conditions, namely the type and dosage of the applied materials, pH, concentration of pollutants, and their appearance form in the medium (i.e., as a single pollutant or as a mixture of PhACs). Among the materials studied, ZnO/Bentonite presented the best performance and resulted in the removal of ~95% of CIP (5 mg/L) in 30 min, at room temperature, near-neutral pH (6.5), ZnO/Bentonite dosage of 0.5 g/L, and under solar light irradiation. The composite also showed a high degree of efficiency for the simultaneous removal of CIP (~98%, 5 mg/L) and SMX (~97%, 5 mg/L) within 30 min, while a low degradation of ~5% was observed for CBZ (5 mg/L) in a mixture of the three PhACs. Furthermore, mechanistic studies using different types of scavengers revealed the formation of active oxidative species responsible for the degradation of CIP in the photocatalytic system studied with the contribution of h^+^ (67%), OH (18%), and ·O_2_^−^ (10%), and in which holes (h^+^) were found to be the dominant oxidative species.

## 1. Introduction

There has been ongoing concern regarding the increased consumption of pharmaceutical active compounds (PhACs) due to their possible accumulation and ecotoxicological impacts [1,2]. They normally originate from various sources such as households, hospitals, and pharmaceutical industries, and their release into receiving environments is significant since most conventional wastewater treatment processes (such as activated sludge) are not designed to control these compounds [3]. Because of their mostly nonbiodegradable character, their accumulation in the environment is expected, especially during pandemic situations (such as COVID-19) [4,5]. To address this issue, several physicochemical and biological methods have been developed and examined in recent years to control PhACs [6,7]. Special attention has been given to advanced oxidation processes (AOPs), which are based on a chain of chemical reactions resulting in the generation of active species (i.e., radical and nonradical oxidizing agents) in the medium for the decomposition of organic compounds [8,9,10]. Various AOPs, such as ozonation [11], catalytic activation of oxidants (such as persulfate (PS), peroxymonosulfate (PMS), iodine, and chlorine) [12,13], electrochemical oxidation [14,15] and photocatalytic processes are among the most widely used AOPs to control various PhACs.

Semiconductor nanomaterials such as TiO_2_ and ZnO have been widely employed for the degradation of various organic compounds using ultraviolet (UV) light [16,17]. There has also been a trend in the literature toward the application of visible-light-active materials produced through tuning the material properties such as band gap energy (BGE) [18,19]. The large BGE of ZnO (approximately 3.37 eV) may render this material a viable alternative for solar light photocatalytic degradation of organic pollutants, especially under high irradiation intensities [20]. However, there are still issues regarding the actual applicability of these materials. The aggregation of particles, when introduced into the aquatic media, can significantly reduce the available surface area and active surface sites and, therefore, can compromise the reactivity of nanomaterials [21]. Stabilization of ZnO nanomaterials using appropriate support materials has been demonstrated to be an efficient way to prevent this issue [22]. Bentonite, being a stable and low-cost clay mineral, with a large surface area and high cation exchange capacity, is considered a suitable alternative to stabilize such semiconductor nanoparticles [23,24]. Difficulties in collecting spent photocatalysts after being used during the treatment process have also been considered an issue that may limit the wider application of such technologies for the treatment of polluted (waste)waters [25]. Adding magnetic properties to photocatalysts has also been studied in recent years as an acceptable way to address this issue [26].

In this manuscript, ZnO and bentonite-supported ZnO (ZnO/Bentonite) nanomaterials were prepared for visible light degradation of PhACs from synthetic effluents. Furthermore, a novel co-loading strategy was adopted through two-stage stabilization of ZnO and γ-Fe_2_O_3_ nanomaterials on the surface of the bentonite to fabricate the ZnO/γ-Fe_2_O_3_/Bentonite composite materials, with the advantage that they can be collected after use in the photocatalytic degradation of PhACs. The mechanisms involved in the removal of the studied pollutants were also studied, in order to provide insights into the elimination pathways of PhACs using this type of photocatalyst.

## 2. Results and Discussions

### 2.1. Characterization of the Prepared Materials

**Figure 1:**
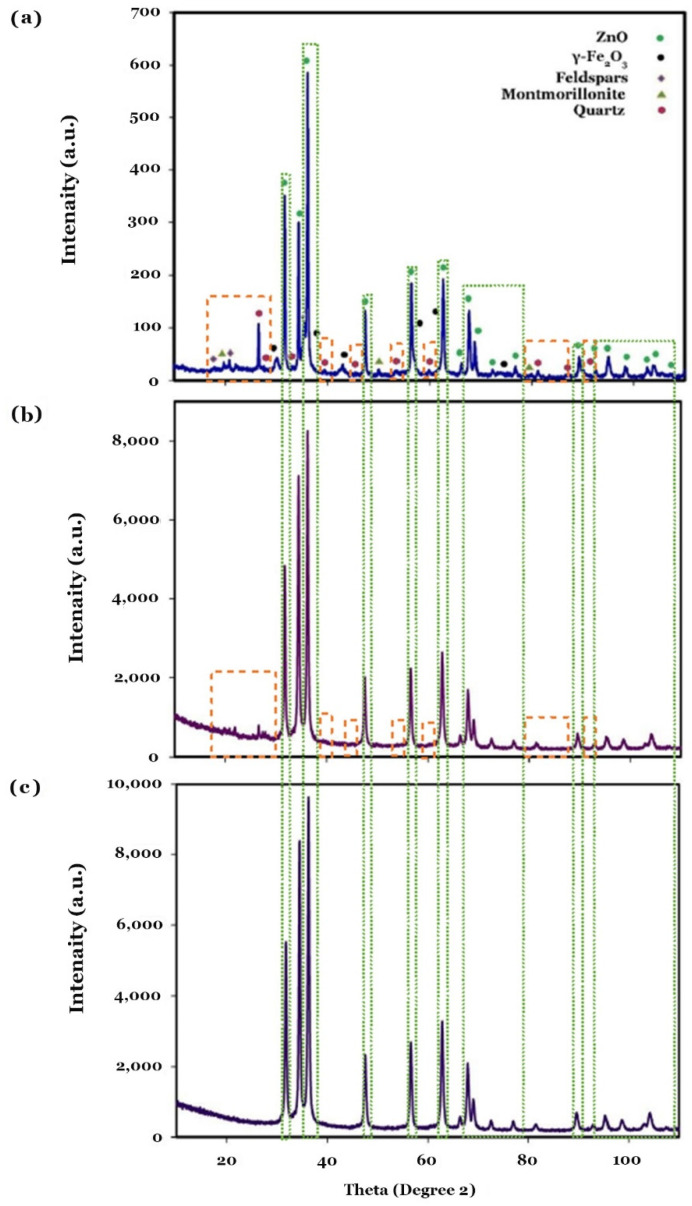
XRD patterns of the samples obtained under a two-step synthesis process for the synthesis of ZnO/γ-Fe_2_O_3_/Bentonite (**a**), ZnO/Bentonite (**b**), and ZnO (**c**).

The XRD patterns of the prepared powders are presented in Figure 1. The identified peaks were indexed with the aid of JCPDS cards. Figure 1a reveals the crystal phase composition of the final product (labeled as ZnO/γ-Fe_2_O_3_/Bentonite), which contains montmorillonite (JCPDS 00-046-1045), quartz (JCPDS 01-077-1060), and feldspar (JCPDS 31-0966) as the main components of natural bentonite [27]) together with ZnO (JCPDS 01-070-8070, zincite) and γ-Fe_2_O_3_ (maghemite, a defective form of magnetite, JCPDS 01-074-0748) evident from the presence of the diffraction peaks, for instance, at 43.28 (2 theta), and 62.92 (2 theta) corresponding to the 400 and 440 diffraction planes of γ-Fe_2_O_3_, which are slightly different from those of magnetite, as previously reported in the literature [28]. Regarding the XRD profile shown in Figure 1b, its peaks were assigned to bentonite and ZnO, demonstrating the formation of ZnO/Bentonite. Finally, all the XRD peaks displayed in Figure 1c were attributed to ZnO, confirming that no secondary phases were precipitated.

**Figure 2:**
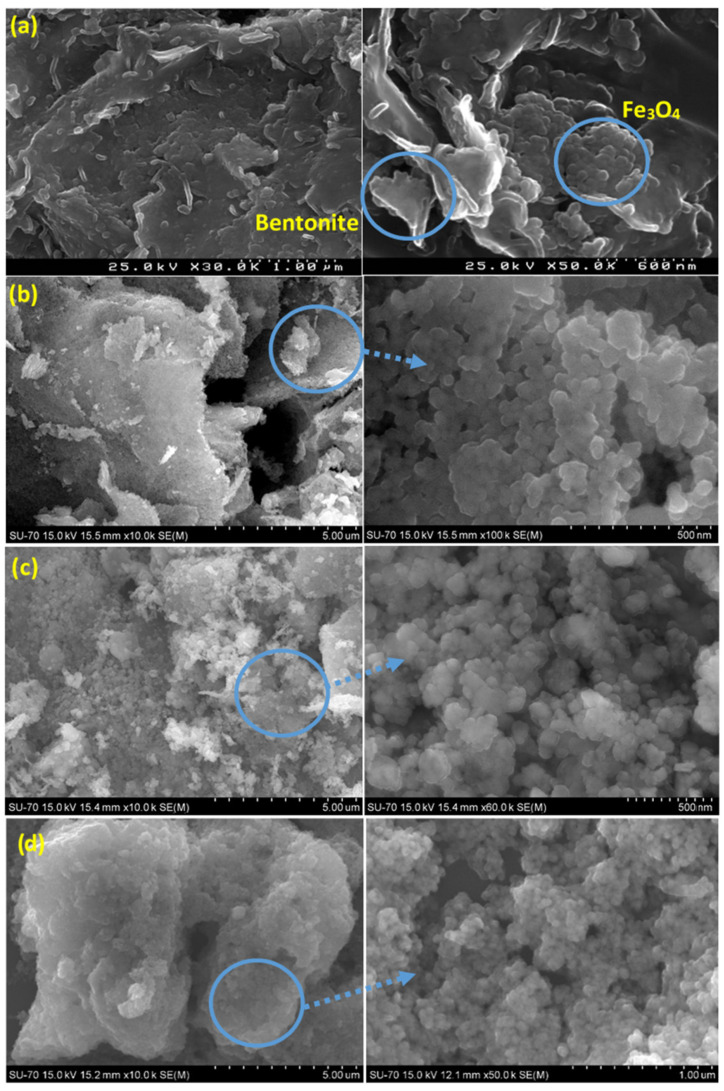
SEM images of Fe_3_O_4_/Bentonite (**a**), ZnO (**b**), ZnO/Bentonite (**c**), and ZnO/γ-Fe_2_O_3_/Bentonite (**d**).

**Figure 3:**
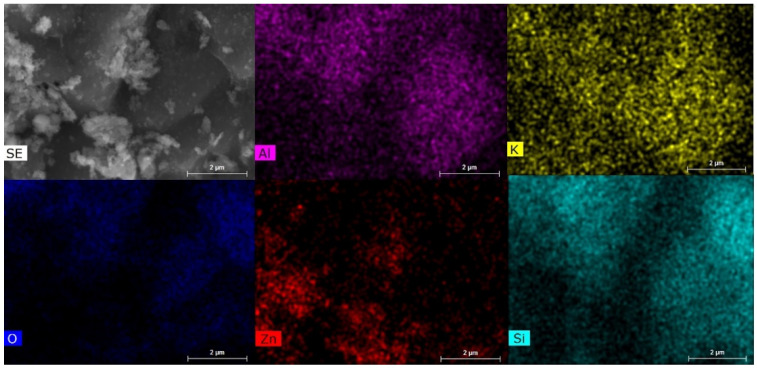
Mapping index of ZnO/Bentonite indicating the presence of essential elements of bentonite (i.e., Al, K, and Si), and Zn conforming to the formation of ZnO in the composite material.

**Figure 4:**
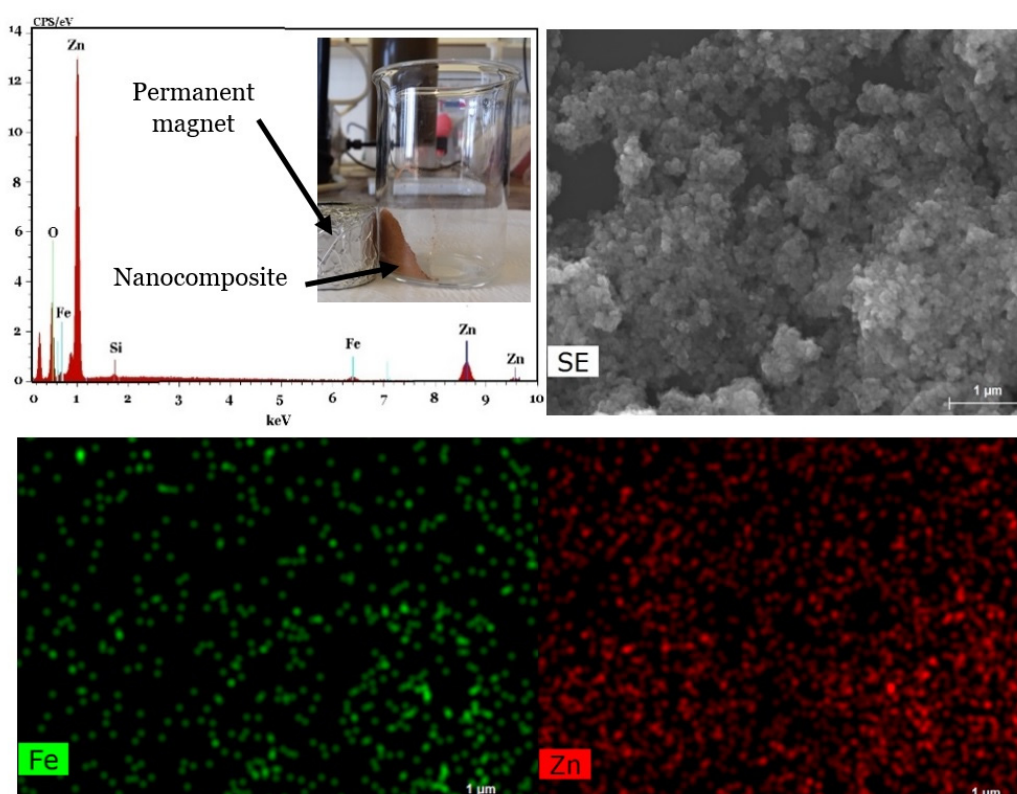
EDS and mapping indexes for Fe and Zn of the sample obtained from the same SEM profile of ZnO/γ-Fe_2_O_3_/Bentonite.

Figure 2 shows the SEM images of the prepared powders, including Fe_3_O_4_/Bentonite (Figure 2a), ZnO (Figure 2b), ZnO/Bentonite (Figure 2c), and ZnO/γ-Fe_2_O_3_/Bentonite (Figure 2d). Figure 2a indicates the growth of spherical Fe_3_O_4_ nanoparticles on the surface of the layered structure of bentonite. Additionally, the precipitated ZnO nanoparticles are spherical, with an average size around 40–50 nm, as observed in Figure 2b. The formation and growth of spherical ZnO nanoparticles on the surface of bentonite are also evident from Figure 2c, being further confirmed by the mapping image of this sample presented in Figure 3. Finally, co-loading of spherical γ-Fe_2_O_3_ and ZnO particles on the bentonite surface is observed in Figure 2d, and the mapping index of this sample is shown in Figure 4, which confirms the presence of Zn and Fe elements.

### 2.2. Photocatalytic Degradation of Pharmaceuticals

#### 2.2.1. Effects of the Operating Conditions

The effects of various operating conditions, including the type and dosage of applied materials (i.e., bentonite or composite photocatalysts), the initial pH of the reaction medium, the type and initial concentrations of the PhACs, and the way pollutants appear in the reaction medium (i.e., as a single pollutant or in a mixture of the three PhACs) on the removal efficiency of PhACs were studied and described in this manuscript.

**Figure 5:**
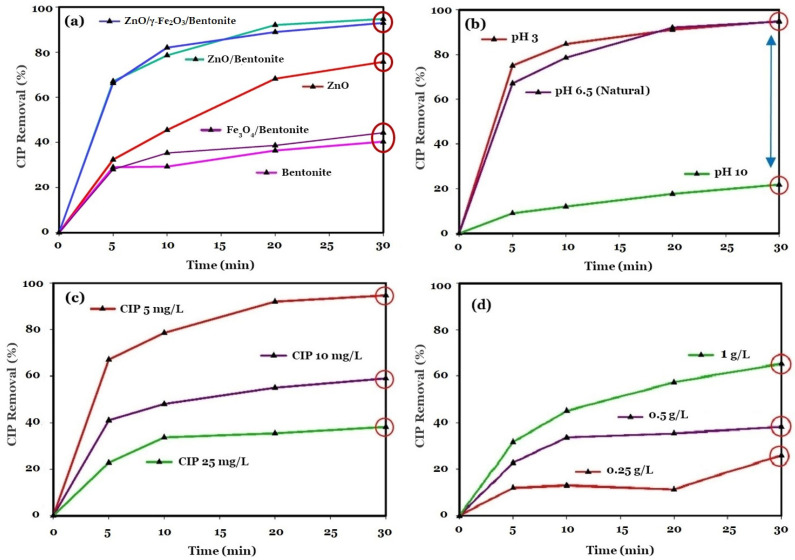
Removal of CIP (5 mg/L) using various types of the applied materials (i.e., bentonite, ZnO/Bentonite, and ZnO/γ-Fe_2_O_3_/Bentonite: 0.5 g/L) under near neutral pH (**a**). Using ZnO/Bentonite (0.5 g/L) under various initial pH conditions (i.e., 3, 6.5, and 10) (**b**). Removal of various initial CIP concentrations (i.e., 5, 10, and 25 mg/L) by ZnO/Bentonite (0.5 g/L) under initial pH 6.5 (**c**). The effects of various ZnO/Bentonite dosage (i.e., 0.25, 0.5, and 1 g/L) were also studied for the removal of CIP (25 mg/L) under near neutral pH conditions (**d**).

The effects of different types of materials (0.5 g/L) applied for the removal of CIP (5 mg/L) are illustrated in Figure 5a. As shown in this figure, bentonite alone resulted in ~40% removal of CIP (*k_obs_* = 0.0133 min^−1^) from the system. Here, it is assumed that adsorption is the main mechanism involved in the removal of PhAC under the cation exchange process, as already discussed and reported in the literature for the adsorption of various organic and inorganic pollutants by clay-based materials such as bentonite [29]. In addition, bentonite-supported magnetite displayed slightly higher removal efficiency for CIP than bentonite, which can be attributed to the effect of magnetite as a known adsorbent for CIP [30]. ZnO alone also resulted in a 75% removal of CIP from the system after 30 min of reaction (*k_obs_* = 0.046 min^−1^) under identical experimental conditions. The use of ZnO-based materials for the photocatalytic degradation of organic pollutants, including PhACs, has already been reported in the literature [31,32,33]. For instance, a 48% removal of CIP (4 mg/L) was observed by El-Kemary et al. (2010) [34] using ZnO nanomaterials after 1 h reaction time under UV irradiation. However, the applicability of ZnO-based photocatalysts under visible light irradiation for the efficient removal of pollutants may facilitate the promotion of the technology for the treatment of actual wastewaters [35]. When loading the ZnO nanoparticles onto the surface of bentonite in the present study (ZnO/Bentonite), an increase in pollutant removal to 95% was observed after 30 min, reaching a *k_obs_* = 0.0949 min^−1^. Co-loading of ZnO and γ-Fe_2_O_3_ (ZnO/γ-Fe_2_O_3_/Bentonite) resulted in ~93% degradation of CIP after 30 min (*k_obs_* = 0.0880 min^−1^). According to these results, optimum performance of the system was achieved using the bentonite-stabilized photocatalysts under the same dosage as for the ZnO assay, and hence a lower ZnO nanomaterial dosage was provided to the reaction medium. This may be associated with the availability of more active sites on the surface of the composite, preventing the rapid agglomeration of particles after being introduced into the medium [21]. Furthermore, bentonite serves as a trap for electrons and leads to limiting the electron-hole recombination rate in the prepared composite materials. In addition, the superoxides formed by the electrons trapped on the surface of bentonite can also further increase the efficiency of the system for the degradation of PhAC [24]. According to the results achieved, the addition of the iron oxide to the composite also adds magnetic properties to the composite, without significantly suppressing the performance of the system to control PhACs. The limited reduction in the efficiency of the system can be explained by the recombination of photoinduced electron-hole pairs in the presence of magnetite, as a well-known conductive material [36].

The effects of the initial pH of the reaction medium on the photocatalytic degradation of CIP are also illustrated in Figure 5b. According to this figure, the lowest degradation efficiency of ~22% was observed at pH 10 with a low *k_obs_* of 0.0076 min^−1^, while both acidic (pH = 3), and near neutral (pH = 6.5) conditions resulted in a high CIP degradation efficiency of about 95%, reaching a k_obs_ of 0.0883 and 0.0949 min^−1^ for pH = 3 and pH = 6.5, respectively. The surface charges of both CIP and ZnO/Bentonite play important roles in the degradation of PhACs under various pH. CIP exhibits various surface charge states, including cationic (pH < 5.9), zwitterionic (5.9 < pH < 8.9), and anionic (pH > 8.9) [37,38]. At pH 10, the electrostatic repulsion between the negative surface charges of both CIP and ZnO/Bentonite hampered the adsorption of CIP onto the surface of ZnO/Bentonite, which resulted in a decrease in the removal efficiency of the system for this PhAC. Hu et al., observed similar results for the photocatalytic degradation of CIP by the TiO_2_-C_3_N_4_ composite, with a decrease in the degradation efficiency from 95% (*k_obs_* = 0.0389 min^−1^) to 40% (*k_obs_* = 0.0162 min^−1^) with increasing pH from 6.3 to 11 [39]. At pH 6.5, CIP represents the zwitterionic form, which facilitates the adsorption of CIP to ZnO/Bentonite through the electrostatic interaction mechanism [40]. In addition, the strong hydrophobic properties of zwitterionic CIP can also improve the degradation efficiency through hydrophobic interactions between the pollutant and the photocatalysts [41].

The efficiency of the ZnO/Bentonite system was also studied for the degradation of different concentrations of CIP, as illustrated in Figure 5c. In this figure, the prepared photocatalyst is shown to represent a high removal efficiency under an initial CIP concentration of 5 mg/L (95% within 30 min, *k_obs_* = 0.0949 min^−1^). However, the efficiency of the system dropped to 59% (*k_obs_* = 0.0251 min^−1^) and 38% (*k_obs_* = 0.0137 min^−1^) by increasing the initial concentration of CIP to 10 and 25 mg/L, respectively. From a mechanistic point of view, the pollutants are first adsorbed on the surface of the composite, especially in the presence of bentonite, after which the surface degradation reactions catalyzed by the photocatalyst compartment of the composite material take place [42,43]. It is suggested that when the initial concentration of the pollutant is 5 mg/L, the extent to which oxidative radicals are produced in the system is sufficient to degrade and remove pollutants already adsorbed on the composite. However, by increasing the concentration of CIP, there is a lack of adequate oxidative species for the removal of pollutants. Furthermore, the system showed a relatively low efficiency for CIP removal with an initial concentration of 25 mg/L, reaching a maximum of 38% degradation after 30 min (*k_obs_* = 0.0137 min^−1^). Here, the amount of oxidative species generated is too low to degrade the pollutant molecules under such high initial concentrations of the pollutant. It is also worth mentioning that the same dosage of composite material was used for different initial concentrations of pollutants. Therefore, at a higher initial concentration of the pollutant, there is limited surface area available for adsorption of the pollutant, and hence, a lower degradation efficiency is present under such conditions. However, under actual conditions, much lower concentrations of PhACs can be expected (from ng/L to µg/L) in most water bodies [44,45,46]. Thus, the utilized technologies can be considered feasible for the removal of PhACs from actual (waste)waters.

The influence of different doses of ZnO/Bentonite (i.e., 0.25, 0.5, and 1 g/L) on the photodegradation of CIP under high initial concentration (25 mg/L) was also studied. The results are illustrated in Figure 5d. According to the results achieved, when increasing the dosage of ZnO/Bentonite from 0.25 to 0.5 and 1 g/L, the efficiency of the system for the degradation of CIP increases substantially from 26% (*k_obs_* = 0.0077 min^−1^) to 38% (*k_obs_* = 0.0137 min^−1^) and 65% (*k_obs_* = 0.0325 min^−1^), respectively. Promoting the efficiency of CIP degradation by increasing the dosage of the composite material used is related to the availability of more active sites for the adsorption of pollutants followed by the photocatalytic degradation of the adsorbed molecules, which results in an improvement in the efficiency of the system [24]. Similar results have been reported in the literature for various AOPs utilizing nanomaterial catalysts [47,48]. However, there are reports available indicating the negative effects of high dosages of photocatalytic materials on the performance of the system, scattering light arrays that can limit the intensity of light reaching the photocatalysts in the medium [49].

**Figure 6:**
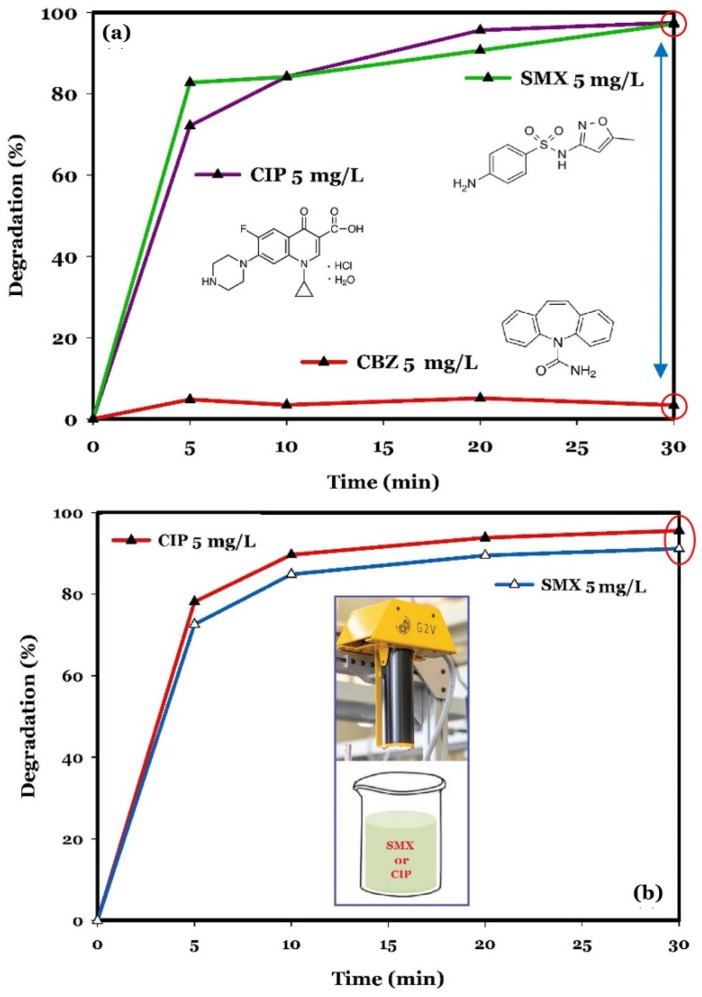
Photodegradation of a mixture of CIP (5 mg/L), SMX (5 mg/L), and CBZ (5 mg/L), using ZnO/Bentonite (0.5 g/L), under near neutral pH and room temperature conditions (**a**), and photodegradation of CIP (5 mg/L) and SMX (5 mg/L), separately, using ZnO/Bentonite (0.5 g/L), under near neutral pH and room temperature conditions (**b**).

To further verify the performance of the treatment system used, different types of contaminants were tested in synthetic effluents using prepared photocatalysts, separately or in a mixture. According to the results achieved, all applied materials (i.e., bentonite, ZnO, ZnO/Bentonite, and ZnO/γ-Fe_2_O_3_/Bentonite) show a low degradation efficiency for the removal of CBZ (<20%). This suggests that there is no significant adsorption and photodegradation of CBZ by the materials studied. The degradation efficiency of a mixture of three pollutants including CIP, SMX, and CBZ was also studied using ZnO/Bentonite. The results are depicted in Figure 6a. The photocatalyst exhibited high degradation efficiencies for CIP (~98%, *k_obs_* = 0.1119 min^−1^) and SMX (97%, *k_obs_* = 0.0979 min^−1^), but only 3% for CBZ (*k_obs_* = 0.0006 min^−1^) has been removed from the system. These results suggest that the structure of CBZ is more stable and complex compared to CIP and SMX, resulting in its high resistance to degradation. As shown in Figure 6a, CBZ has three phenolic rings in its molecular structure, while the two rings of CIP molecules are linked by a relatively weak covalent bond (N-C). Furthermore, SMX has only a single phenolic ring in its molecular structure. Hence, it can be speculated that the CBZ resists decomposition more, compared to both CIP and SMX. There are some reports in the literature that support this idea. For instance, Che et al. [50] observed that the CIP and tetracycline (TC) degradation efficiencies reach 50% and 90%, respectively, with a photocatalytic system using CdS/Bi_3_O_4_Cl. Based on their proposed degradation pathways, ring-opening and oxidation reaction are the first steps involved in the degradation of CIP and TC in such a system, respectively. Ring-opening reactions require more activated species, resulting in a decrease in the degradation efficiency of the AOP applied to control more complex compounds.

The results achieved in the present study also indicated a high degradation efficiency of SMX (91%, *k_obs_* = 0.0698 min^−1^) when added separately to the synthetic effluents, very close to the degradation efficiency of CIP (95%, *k_obs_* = 0.0949 min^−1^), both with ZnO/Bentonite, as demonstrated in Figure 6b [51,52,53].

#### 2.2.2. Mechanistic Studies

To investigate the mechanisms involved in the photocatalytic degradation of CIP using ZnO/Bentonite, different scavenging agents were added to the synthetic effluents before adding the photocatalyst. In practice, triethanolamine (TEOA), benzoquinone (BQ), tertiary butanol (t-BuOH), and ethanol were used as scavengers for holes (h^+^), superoxide radical anions (·O_2_^−^), hydroxyl radicals (·OH), and both electrons (e^−^) and hydroxyl radicals (·OH), respectively [54,55,56].

**Figure 7:**
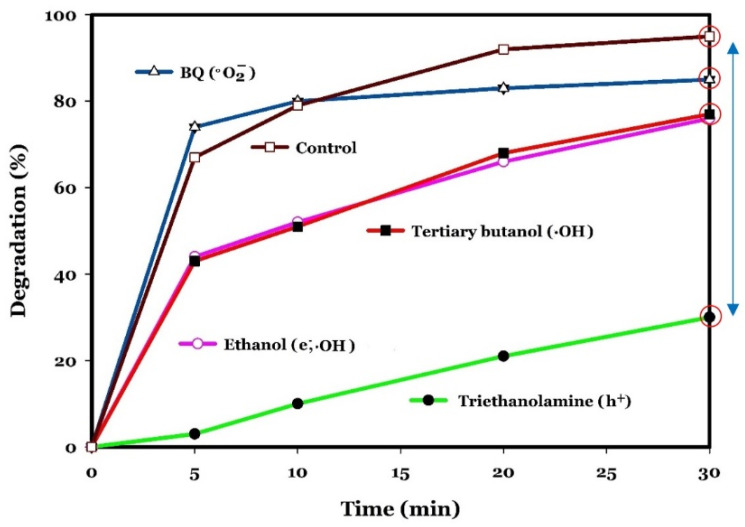
Decreased photocatalytic degradation of CIP (5 mg/L) using ZnO/Bentonite (0.5 g/L), as a result of the addition of different scavenging agents.

**Figure 8:**
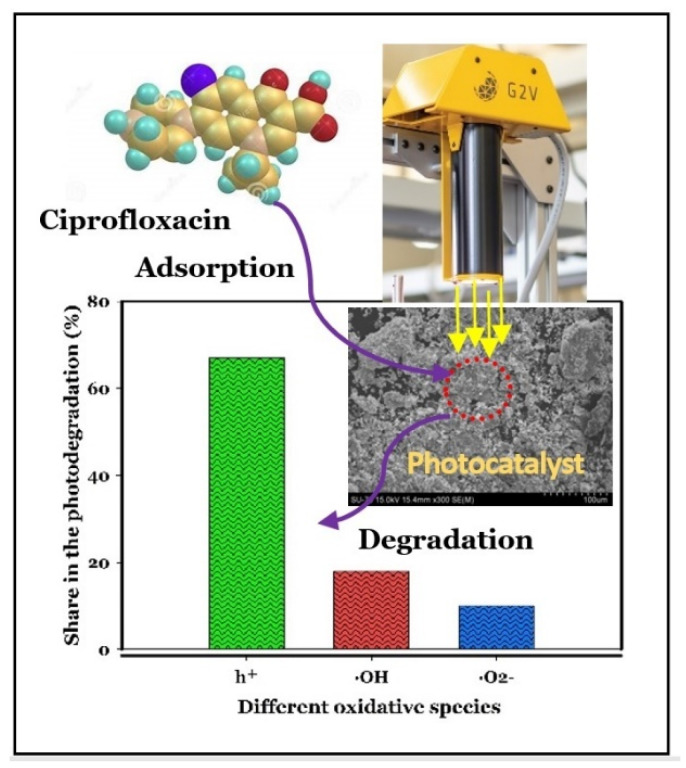
Contribution of various oxidative species in the photodegradation of CIP (5 mg/L) using ZnO/Bentonite (0.5 g/L).

The scavenging experiments were carried out to reveal the contribution of the various active species to the CIP degradation. The results are presented in Figure 7. According to this figure, the removal rate of CIP was significantly inhibited by the addition of TEOA, suggesting that h^+^ radicals are the dominant reactive species in the photocatalytic degradation process using ZnO/Bentonite. Additionally, only a slight decrease in the removal of this pollutant was observed when t-BuOH was added to the reaction system, revealing that ·OH was also involved, but with a marginal role in the photocatalytic degradation of CIP. Furthermore, a negligible effect of CIP degradation was identified in the presence of BQ, implying that ·O_2_^−^ radicals were not the crucial species for CIP degradation, but had a minor role in the photocatalytic degradation process. Additionally, the results of the scavenging experiments with t-BuOH and ethanol revealed that e^−^ has no significant effects on the direct decomposition of CIP. The contribution of different oxidative species to the photodegradation of CIP under this system is presented in Figure 8.

Based on the oxidative species identified, a possible mechanism for the CIP degradation is proposed, according to Equations (1)–(7). Under solar irradiation, electrons and holes are formed in the conduction band and in the valance band of the ZnO, respectively (Equation (1)). Photogenerated electrons can react with dissolved oxygen to produce ·O2^−^ (Equation (2)), as reported in the literature [57]. Simultaneously, the reaction between the water molecules and holes results in the generation of ·OH, according to Equation (3). The generated h^+^ can further react with ·O_2_^−^ to produce hydrogen peroxide (Equation (4)). H_2_O_2_ molecules are very reactive with electrons to form ·OH and hydroxide ion species (OH^-^), as indicated in Equation (5). The OH^−^ produced can also react with h^+^ to produce ·OH (Equation (6)) [57,58,59]. The resulting active species, including h^+^, ·OH, and ·O_2_^−^, contribute to the oxidization of CIP to end products (water and carbon dioxide), or to degradation byproducts that remain in the reaction medium (Equation (7)).

ZnO/Bentonite + hv → h^+^ + e^−^ (1)

O_2_ + e^−^ → ·O_2_^−^ (2)

h^+^ + H_2_O → ·OH + H^+^ (3)

2H^+^ + ·O_2_^−^ + e^−^ → H_2_O_2_ (4)

H_2_O_2_ + e^−^ → ·OH + OH^−^ (5)

h^+^ + OH^−^ → ·OH (6)

h^+^, ·OH, ·O_2_^−^ + CIP→ degradation by- and/or final products (7)

## 3. Materials and Methods

### 3.1. Chemicals

Ferric chloride (FeCl_3_·6H_2_O, ACS reagent, 97%) and ferrous sulfate (FeSO_4_·7H_2_O, analytical reagent) were purchased from Sigma-Aldrich (Overijse, Belgium) and Fisher Chemical (Loughborough, UK), respectively, and used together with aqueous ammonia (NH_3_H_2_O, 28–30%, Acros Organics, Geel, Belgium) for the synthesis of Fe_3_O_4_ nanomaterials. In addition, zinc acetate (ZnAC_2_.2H_2_O, ACS reagent, ≥98%, Sigma-Aldrich, Overijse, Belgium) and ammonium carbonate ((NH_4_)_2_CO_3_, analytical reagent, Santa Cruz Biotechnology, Heidelberg, Germany) were used for the preparation of ZnO nanomaterials, and bentonite (analytical reagent, Chem-Lab, Zedelgem, Belgium) was used for the stabilization of nanomaterials. Carbamazepine (CBZ, analytical grade, ≥98%, Sigma-Aldrich, Darmstadt, Germany), ciprofloxacin (CIP, analytical grade, ≥98%, Sigma-Aldrich, Darmstadt, Germany) and sulfamethoxazole (SMX, >98%, TCI Europe, Zwijndrecht, Belgium) were also used to prepare the synthetic effluents. Chemicals including triethanolamine (≥98%, Chem-Lab, Zedelgem, Belgium), benzoquinone (99%, Acros Organics, Geel, Belgium), tertiary butanol (98%, Fisher Chemical, Loughborough, UK), and ethanol (99% Fisher Scientific, Loughborough, UK) were used for the scavenging experiments. In addition, ammonium formate (NH_4_HCO_2_, Acros Organics, Geel, Belgium), formic acid (CH_2_O_2_, >98%, Sigma-Aldrich, Overijse, Belgium), and acetonitrile (Sigma-Aldrich, Saint Louis, MO, USA) were used in the high-performance liquid chromatography (HPLC) analysis.

### 3.2. Preparation of the Nanomaterials

#### 3.2.1. Synthesis of Fe_3_O_4_/Bentonite

Fe_3_O_4_/Bentonite nanomaterials were synthesized using a facile co-precipitation method. In a typical synthesis process, 0.61 g FeCl_3_·6H_2_O and 0.42 g FeSO_4_·7H_2_O were mixed in 100 mL of deionized water with 1 g of bentonite under continuous stirring using a mechanical stirrer (100 rpm) at 35 °C. After stirring for 10 min, 10 mL of ammonia (30%) was added dropwise to this solution. Stirring was continued for 30 min at 35 °C to complete the synthesis reactions. Finally, the black precipitates were collected using a permanent magnet and washed three times with deionized water to remove impurities. The precipitate obtained was named Fe_3_O_4_/Bentonite.

#### 3.2.2. Synthesis of ZnO/γ-Fe_2_O_3_/Bentonite

The material prepared as described in Section 2.2.1. (i.e., Fe_3_O_4_/Bentonite) was dispersed in 100 mL deionized water and stirred under ultrasonic irradiation for 10 min. Then, 6.08 g of zinc acetate dihydrate and 3.8 g of ammonium carbonate were dissolved separately in 100 mL of deionized water. The two solutions were added to the reactor containing the suspension of Fe_3_O_4_/Bentonite. The final precipitate was then collected and washed several times with distilled water and ethanol to remove impurities. Finally, the prepared material was calcinated in a muffle furnace at 350 °C for 3 h. The resulting powders were labeled as ZnO/γ-Fe_2_O_3_/Bentonite and collected for characterization. ZnO and ZnO/Bentonite powders were also synthesized using the same method without Fe_3_O_4_/Bentonite or using only bentonite instead of Fe_3_O_4_/Bentonite.

### 3.3. Characterization

The crystalline structure of the prepared powders was explored using X-ray diffraction (XRD) pattern analysis (XRD, Panalytical X’Pert PRO 3, Almelo, The Netherlands). Additionally, the morphology and elemental composition of the particles were characterized using a scanning electron microscope (SEM, Hitachi SU-70, Tokyo, Japan).

### 3.4. Degradation Experiments

Photocatalytic degradation experiments were investigated through the addition of the prepared materials into a 250 mL reactor containing 100 mL of PhACs (i.e., CIP, CBZ, SMX). The solution pH was adjusted by the addition of 0.1 M HCl or 0.1 M NaOH, when necessary. The reactor was placed under a G2V Small Area Pico Solar Simulator with a light intensity of 79.4 mW/cm^2^ under continuous stirring at 100 rpm. At regular time intervals, 2 mL of solution was filtered through a 0.45 μm filter membrane and then the concentration of the PhACs was measured using an HPLC as described in the next section. The degradation efficiency of the system was calculated according to Equation (8):

Degradation (%) = 100 × ((C_0_ − C_t_)/C_0_) (8)

where *C_0_* and *C_t_* are the pollutant concentration at the initial time and at the sampling time (t), respectively. A pseudo-first-order model was also fitted to the kinetics of the reactions involved in the photocatalytic degradation of PhACs (Equation (9)):

(9) $$\frac{d\left[\mathrm{PhACs}\right]}{\mathrm{dt}}=-k_{obs}\times {\left[\mathrm{PhACs}\right]}_{t}$$

Or:

(10) $$\ln\frac{{\left[\mathrm{PhACs}\right]}_{t}}{{\left[\mathrm{PhACs}\right]}_{0}}=-k_{obs}\times t$$

In these equations, *k_obs_* is the pseudo-first-order reaction rate constant (min^−1^), calculated as the slope of the fitted linear regression between ln([PhACs]_t_/[PhACs]_0_) and the reaction time (t). In addition, [PhACs]_0_ and [PhACs]_t_ are the initial concentration of the PhACs and their concentration at time t, respectively [27]. The possible mechanisms involved in the degradation of CIP using photocatalysts were investigated using scavenging experiments. For this, triethanolamine, benzoquinone, tertiary butanol, and ethanol were used as 4 scavengers for holes (h^+^), superoxide radical anions (·O_2_^−^), hydroxyl radicals (·OH), and both electron (e^−^) and hydroxyl radicals (·OH), respectively [54,55,56].

### 3.5. Analytical Methods

The concentrations of the contaminants studied were analyzed by an Agilent 1100 HPLC system, including a quaternary pump, an autosampler, and a VWD detector. For the CIP, a C18 column (Zorbax Eclipse Plus, 4.6 × 100 mm; particle size (dp): 3.5 μm) and UV-detection wavelength of 280 nm were used. In a typical process, 10 mM ammonium formate was dissolved in water and the pH adjusted to 2.8 with formic acid to form solution A. This solution together with acetonitrile (ACN) (solution B) were used as mobile phases. The elution method consisted of a gradient, starting from 10% B, and increasing linearly to 15% B in 15 min. The column was reconditioned for 5 min at the initial conditions. The flow rate and the injection volume were 1.0 mL/min and 3 μL, respectively. For the CBZ, a C18 column (Agilent Eclipse Plus, 4.6 × 250 mm, dp = 5 μm) was used. The mobile phase included water and ACN with a ratio of 60:40 (*v*/*v*). The UV detector and the sample injection volumes were 284 nm and 30 μL, respectively. For the SMX, a C18 column (Zorbax Eclipse Plus, 4.6 × 100 mm; particle size (dp): 3.5 μm) was used. The mobile phases included water with 0.1% formic acid (solution A) and ACN with 0.1% formic acid (solution B). The elution method consisted of a gradient, increasing from 10% B to 95% B in 15 min, and maintained at 95% B for 10 min, then returned to the initial conditions in 10 min. The flow rate, injection volume, and UV-detection wavelength were 1 mL/min, 3 μL, and 280 nm, respectively.

## 4. Conclusions

This study reports a novel two-step co-loading strategy for the synthesis of ZnO/γ-Fe_2_O_3_/Bentonite. ZnO/Bentonite and ZnO nanoparticles were also prepared for solar-light photocatalytic degradation of PhACs. Both ZnO/γ-Fe_2_O_3_/Bentonite and ZnO/Bentonite showed superior removal efficiency (i.e., 93%, and 95%, respectively), compared to ZnO alone and bentonite alone (i.e., 75%, and 40%, respectively) after 30 min of reaction and under simulated solar irradiation and near-neutral pH conditions. This can be attributed to the stabilization of the nanoparticles on the layered structure of bentonite, which can prevent the agglomeration of ZnO nanomaterials, providing more active sites on the surface of the composites. This strategy can also lead to a decrease in the recombination rate of electrons and holes, resulting in the promotion of the efficiency of the system. The results also indicated the significant roles of operating conditions, including photocatalyst dosage, operating pH, and the type and concentration of pollutants. Finally, scavenging experiments confirmed the formation of active oxidative species that contribute to the degradation of CIP in the photocatalytic system studied, with a contribution of h^+^ (67%), ·OH (18%), and ·O_2_^−^ (10%),), and in which holes (h^+^) were found to be the dominant oxidative species.

## Data Availability

Not applicable.

## Sample Availability

Samples of the compounds are available from the authors.
